# A qualitative study of how clinicians reach agreement in perioperative pathway development: the Consensus Model for Standardising Healthcare

**DOI:** 10.1186/s43058-025-00699-9

**Published:** 2025-02-04

**Authors:** Lisa Pagano, Janet C. Long, Emilie Francis-Auton, Andrew Hirschhorn, Gaston Arnolda, Jeffrey Braithwaite, Mitchell N. Sarkies

**Affiliations:** 1 Australian Institute of Health Innovation, Faculty of Medicine, Health and Human Sciences, Macquarie University, Sydney, Australia; 2 MQ Health, Faculty of Medicine, Health and Human Sciences, Macquarie University, Sydney, Australia; 3 School of Health Sciences, Faculty of Medicine and Health, University of Sydney, Sydney, Australia; 4 Implementation Science Academy, Sydney Health Partners, University of Sydney, Sydney, Australia

**Keywords:** Implementation science, Consensus, Implementation, Strategy, Mechanism, Perioperative care

## Abstract

**Background:**

Variation in perioperative care persists globally. Consensus discussions may facilitate standardisation, yet the processes used to reach agreement are poorly understood. This study aimed to develop a model for conducting local consensus discussions when implementing standardised perioperative pathways. Specifically, we 1) describe how local consensus discussions are operationalised; 2) identify what guides decision making and consensus between clinicians; and 3) formulate explanatory mechanisms and identify determinants that facilitate consensus discussions.

**Methods:**

A qualitative, modified grounded theory study was conducted in one private hospital in metropolitan Sydney, Australia. Thirty-one participants from clinical disciplines and hospital management/leadership were included. Data were collected from nine semi-structured interviews and 16 h of participant observations during consensus development or implementation meetings. Data collection and analysis occurred concurrently until theoretical saturation was achieved. Interviews and field notes were recorded and transcribed verbatim. Data were analysed using coding, constant comparison, detailed memo writing and data interpretation.

**Results:**

Seven individual and contextual factors crucial for building consensus, and eight mechanisms for reaching agreement were identified and integrated into a conceptual model. Seeking evidence to support decision-making emerged as the primary driver of consensus. Strong research evidence in support of a pathway component facilitated swift agreement. Where there was ambiguous evidence for a pathway component, clinicians based their decisions on a desire for professional autonomy, consideration of how their peers practice, patient preferences, practices from external organisations, or the feasibility of implementing the pathway component.

**Conclusions:**

The Consensus Model for Standardising Healthcare provides a map for healthcare organisations seeking to conduct local consensus discussions to reduce variation in care. Our findings advance our understanding of how local consensus discussions are conducted and factors that impact success when standardising care amongst clinicians.

**Supplementary Information:**

The online version contains supplementary material available at 10.1186/s43058-025-00699-9.

Contributions to the literature

- This study addresses recognised gaps in the literature, including how decisions are commonly made in healthcare settings when using the implementation strategy ‘conducting local consensus discussions’.
- We developed a model highlighting key factors in consensus-building in healthcare showcasing the key steps and contextual factors that impact on local consensus discussions, which advances our understanding of how consensus processes can be used in healthcare settings to standardise care practices.
- We identified key mechanisms that drive clinicians toward agreement, with evidence-seeking as the primary driver.
- Mechanisms varied in importance, forming a hierarchy in their influence on consensus.

## Introduction

Health professionals make many clinical decisions daily. When these decisions are made in silos, unwarranted variation in care is more likely to occur [1, 2]. Unwarranted clinical variation refers to patient care that differs in ways that are not a direct and proportionate response to available evidence, or to the healthcare needs and informed choices of patients [3, 4]. It is estimated that some 30% of care delivered is considered low value [5, 6], which can exacerbate disparities in patient outcomes and contribute to increased healthcare costs [7]. In surgery, substantial international variation in perioperative care has been reported, which encompasses the care patients receive from the moment of contemplation of surgery until full recovery [8, 9]. For example, data from Australia and the United States reveal significant variation in length of stay, rates of transfer to inpatient rehabilitation, and higher rates of unplanned stays over two hours in recovery after hip and knee arthroplasty [8, 10, 11]. In some cases, this variation occurs despite the presence of abundant, high-quality evidence such as through evidence-based guidelines, where their inclusion in clinical practice is not automatically guaranteed [12]. Reducing unwarranted clinical variation has become a priority in surgical and perioperative care to ensure that care delivery is effective, efficient and timely [7, 13].

Surgeons and proceduralists highly value professional autonomy in their practice, relying on evidence in conjunction with expert opinion, patient-centred care and individual experience [2, 14, 15]. While this autonomy can prioritise individualised patient care, it may also contribute to variation in both processes and outcomes that can lead to gaps in care quality. To reduce variation, perioperative pathways are increasingly being used to standardise care across the pre-operative, intraoperative and postoperative stages of the patient journey [16]. Perioperative pathways, such as Enhanced Recovery After Surgery protocols [17], have demonstrated benefits in reducing hospital costs and length of stay, while maintaining positive outcomes for patients [18]. Further, whilst finding the optimal balance or potential synergy between standardised and personalised care can be challenging for healthcare practitioners, there is growing evidence that adhering to standardised guidelines can improve patient experience [19–21]. However, implementing standardised pathways poses unique challenges, necessitating multi-clinician and multidisciplinary efforts to obtain agreement from many stakeholders with diverse preferences [22].

Implementation strategies aimed at fostering agreement between stakeholders are crucial to gain buy-in and standardise practices. Conducting local consensus discussions is a strategy which supports clinicians in reaching agreement and has demonstrated success in overcoming barriers related to communication within organisations, designing and assembling an intervention and promoting leadership engagement in implementation trials [23, 24]. However, there is limited documentation on how this strategy is used in real-world clinical practice, as studies have primarily focussed on the outcomes of consensus discussions rather than the methods of operationalising them. Developing consensus is difficult when we have limited understanding of the mechanisms by which agreement is sought in real world practice.

Much of our understanding of consensus methods are rooted in evidence on formal processes, such as the Delphi method in clinical guideline development and Nominal Group Technique in the prioritisation of evidence gaps [25]. However, restricting our understanding of consensus discussions to formal methods is limiting due to the fact that many consensus processes do not follow these methods, reflecting the time and resource demands involved. Healthcare organisations routinely undertake informal consensus methods led by clinicians, where stakeholders might set their own ground rules for behaviour and decision-making [26–28].

The effectiveness of local consensus discussions in developing perioperative pathways and reducing unwarranted clinical variation likely depends on how the strategy is utilised and its interaction with contextual factors. Research in other fields such as law and business have derived theories of consensus-building that describe the process of seeking unanimous agreement by making a good faith effort to address the interests of all stakeholders, achieving a solution that everyone can accept [26, 29, 30]. This literature posits that several conditions must be present for a process to be labelled as consensus-building [26] yet, it often stops short of specifying a sequence of steps. Each context is unique and requires tailored strategies, particularly when developing interventions like perioperative pathways that require agreement from numerous individuals [31, 32]. Mechanistic understanding is essential for designing interventions that are both theoretically sound and practically effective in diverse healthcare settings [33]. To further our ability to effectively use consensus discussions in healthcare an understanding of the determinants impacting the success of this strategy and the mechanisms driving consensus discussions and how clinicians reach agreement is required.

### Aims

The aim of this research was to develop a model for conducting local consensus discussions when implementing standardised perioperative pathways. Our objectives were to:

1. Describe how local consensus discussions are operationalised.
2. Identify what guides decision making and the criteria for consensus between clinicians.
3. Formulate explanatory mechanisms that drive consensus and the determinants that facilitate consensus discussions to develop perioperative pathways.

## Materials and methods

A qualitative study using semi-structured interviews and participant observation was conducted using a constructivist, modified grounded theory approach between February 2023 to May 2024. A constructivist approach is interpretive in nature, viewing research interactions as opportunities for co-creating knowledge that can provide deeper insights into experiences, and highlights how participants’ perspectives and researcher insights collectively shape the findings [34]. Where classical grounded theory asserts that theory emerges only from the data, a constructivist approach acknowledges the role of the researcher in data generation [35]. It is useful for capturing the complexity of social interactions and decision-making processes through interviews and observations, as it allows for flexibility in adapting to emerging themes and patterns during data collection [36, 37].

The manuscript follows the Standards for Reporting Qualitative Research (SRQR) checklist (Supplementary File 1). This study received ethical approval from (details anonymised).

### Study setting and intervention

This qualitative study was embedded within a larger observational study [38]. The study site was a university-owned, teaching hospital located in metropolitan Sydney, Australia. This hospital is a privately funded 144-bed facility, including a 20-bed intensive care unit, that focusses on clinical care, teaching and research.

The purpose of this larger observational study was to examine the process of developing and implementing perioperative pathways for four surgical cohorts using an organisationally supported consensus approach. The initiative was driven by the desire to improve patient outcome metrics in line with peer organisations and to streamline care processes for specific surgical cohorts. The initial surgical cohorts included i) total hip and knee arthroplasty; ii) spinal surgery and; iii) cardiothoracic surgery. Breast cancer surgery (iv) was included as an additional cohort following publication of the protocol [38].

The consensus approach involved conducting local consensus discussions between cohort-specific groups of stakeholders, comprising multidisciplinary representation from clinical, non-clinical and leadership disciplines. Clinical consensus groups attended regular meetings facilitated by a clinician–researcher to discuss and agree on components of care to be included in the standardised perioperative pathways, using an informal consensus approach. Consensus on an implementation plan was also achieved in separate meetings, which included additional frontline clinicians and members of the consensus groups. Updates on the project’s progress and presentation of the finalised pathways were reported at leadership meetings and stakeholder engagement meetings to engage frontline clinical staff in implementation.

### Participants and sampling

Convenience sampling was used to recruit for the participant observation component of this study. Hospital staff involved in consensus discussions or relevant meetings were invited to participate by hospital coinvestigators from the research team after being informed about the study. Participants could opt-out of participating in the observations at any time throughout the study period.

Concurrent to the participant observations, a sample of local hospital staff involved in the delivery of clinical care for the specified surgical cohorts or those involved in consensus discussions were invited to participate in semi-structured interviews. Staff in clinical, non-clinical and leadership roles were considered eligible to ensure the selection of especially knowledgeable and ‘information-rich’ cases about all aspects of developing or implementing the pathways [39].

Participants were invited by a hospital-based co-investigator who were then sent follow-up information by a member of the research team via email. Prior to the interviews and participant observations, participants were provided with an information form, and verbal or written informed consent was obtained prior to participation.

### Data collection

Naturalistic participant observations were conducted of consensus discussions or meetings relating to the development or implementation of perioperative pathways. A hospital-based co-investigator identified relevant and suitable meetings for observation for the research team to attend. Observations were conducted by one researcher (LP) with experience in conducting qualitative research in healthcare. Field notes were taken in real time to document the spatial, social and temporal context of the meetings and more detailed typed field notes were completed following the observations. While the researcher was always cognisant of study aims and objectives during the observation process, observations were not guided by a pre-determined schedule or prepared tools. Ideas evolved inductively with what was emerging within each context, as is consistent with a grounded theory approach [37].

Individual, face-to-face semi-structured interviews were conducted by the same researcher (LP) in quiet, private locations that were convenient for the participant. Interviews followed one of three general interview schedules which were designed for each sub-group: i) surgeons (Supplementary File 2); ii) nursing or allied health (Supplementary File 3) and iii) management or leadership (Supplementary File 4). Questions were open-ended with flexibility in the order and wording of questions and probes or additional questions were used to clarify statements where necessary. Each interview took approximately 30–60 min to complete. The interviewer wrote case-based memos during and following each interview which included initial thoughts, interpretations and analyses of the data collected. All interviews were digitally recorded and transcribed verbatim in preparation for analysis. An audit trail of methodological decisions made during research were recorded. Observations and interviews were conducted in two phases; to first develop preliminary interpretations and to then test and refine the initial interpretations. Data collection was discontinued when theoretical saturation had been achieved.

### Data analysis

Data collection and analysis occurred concurrently. Ground theory methods were used for analysis including coding, constant comparison, memo writing and theoretical sampling. These methods not only help researchers to synthesise data but assist them to move beyond description through to constructing new concepts that explain what is happening [34]. Transcripts and observation field notes were analysed using the software NVivo, V14. Transcripts were read numerous times to ensure immersion prior to coding. Inductive, line-by-line coding was first conducted with constant comparison to other data segments and codes on three transcripts by one researcher (LP). This was then discussed with two qualitative researchers (JL, MS) to create the initial coding framework and subsequent coding was continued by one researcher (LP), supported by team meetings and discussions with the core research team where researchers compared their interpretations (LP, JL, EF-A, MS). Data from interviews and observations were triangulated to allow for a more comprehensive understanding of the research question through the convergence of multiple data sources and perspectives. The most frequent and significant codes from the initial coding framework were synthesised into focussed codes and categories to identify emerging concepts (see Supplementary File 5). Discussion of these focussed codes and generation of preliminary relationships between the codes supported the creation of a preliminary model. Conceptual memos about codes were also recorded, coded and analysed [34].

Initial interpretations were tested in subsequent interviews and observations. Interview guides for these interviews were also revised and refined to include more focussed questions relating to the core concepts emerging from the data; for example, questions relating to each mechanism of consensus initially identified. We then conducted theoretical coding where the relationships between each core concept/category from our initial data analysis were further examined and integrated into theoretical concepts, designed to ‘give integrative scope, broad pictures and a new perspective’ [40, 41]. The data was then used to refine the model.

## Results

The research team attended every observation opportunity to which they were invited. Approximately sixteen hours from 15 meeting observations with staff from clinical, non-clinical and leadership roles (n = 31) were conducted. All staff present at the meetings consented to be observed. Seventeen staff members were identified by the clinician-researcher as suitable to participate in a semi-structured interview and were subsequently invited. Of these, two individuals declined to participate and seven did not respond. Nine individual interviews were completed, with one individual interviewed twice for theoretical validation purposes. The characteristics of participants and each observation can be found in Tables 1 and 2.

**Table 1:** Characteristics of participants

**Item**	**Number (%)**
***Observation participants***	***n* = *31***^***a***^
**Discipline**	
Surgeon	7 (23%)
Anaesthetist	2 (6%)
Nursing	7 (23%)
Nursing education	3 (10%)
Nursing unit manager	6 (19%)
Physiotherapist	2 (6%)
Leadership/management	4 (13%)
**Gender**	
Male	15 (48%)
Female	16 (52%)
***Interview participants***	***n* = *8***^***b***^
**Discipline**	
Anaesthetist	1 (13%)
Nursing	2 (25%)
Physiotherapist	1 (13%)
Leadership/management	4 (50%)
**Gender**	
Male	2 (25%)
Female	6 (75%)
**Number of years worked at organisation**	
< 5 years	3 (38%)
6–10 years	4 (50%)
11 + years	1 (13%)
**Number of years worked in healthcare**	
10–20 years	3 (38%)
20–30 years	2 (25%)
30–40 years	2 (25%)
40 + years	1 (13%)

^a^22 participants observed on more than one occasion

^b^A total of nine interviews were completed with one participant interviewed on two occasions

**Table 2:** Data observation sources

**Meeting category**	**Number (%), *n* = 15**
**Consensus meeting for pathway development**	
Total hip and knee arthroplasty	2 (13%)
Spinal surgery	3 (20%)
Cardiac surgery	3 (20%)
Breast cancer surgery	2 (13%)
**Consensus meeting for implementation planning**	
Total hip and knee arthroplasty	1 (7%)
Spinal surgery	2 (13%)
**Nursing committee and/or leadership meetings**	2 (13%)

**Fig. 1:**
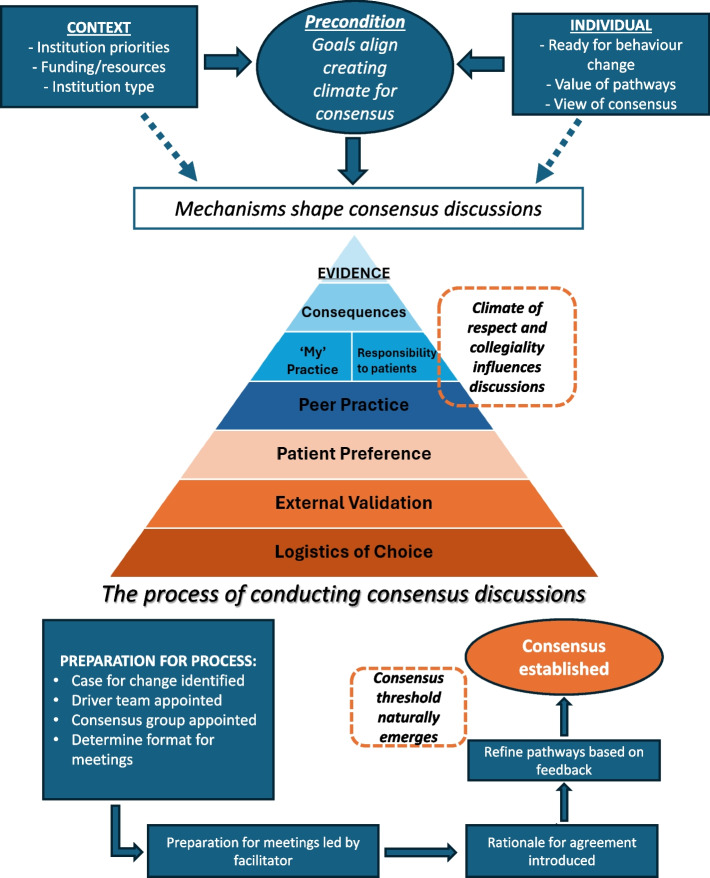
The Consensus Model for Standardising Healthcare: the conceptual model demonstrates the key concepts involved in ‘conducting local consensus discussions’ including; i) sequence of steps in conducting local consensus discussions; ii) determinants comprising what we refer to as the ‘consensus climate’ which influences the success of progressing through each step, and; iii) explanatory mechanisms revealing how participants approached reaching consensus

We developed a conceptual model for conducting local consensus discussions: The Consensus Model for Standardising Healthcare (Fig. 1). Supplementary File 6 provides illustrative quotes pertaining to each concept in the model. The Consensus Model for Standardising Healthcare describes how local consensus discussions were operationalised through a series of key steps. The overall success of consensus discussions was influenced by determinants at the individual and organisational levels which collectively shaped the consensus 'climate'. Achieving consensus was driven by various mechanisms, facilitating the natural emergence of a threshold for agreement. How these determinants and mechanisms influence or mediate the consensus process is illustrated by nine hypotheses generated from our data (Table 3).

### The consensus discussion process

The process of reaching consensus began with identifying the motivations of the organisation and stakeholders to engage in a consensus process to develop perioperative pathways. Subsequently, a 'driver' team was appointed, comprising leadership, management, and clinical disciplines. Participants emphasised the importance of including an internal facilitator within the driver team who was familiar with the organisation's dynamics and possessed extensive experience within the organisation. Consensus group composition was determined either by the driver team or by clinicians with relevant expertise (usually surgeons, anaesthetists or nursing leaders). Broad disciplinary representation was recognised as essential for successful consensus-building, ensuring input was obtained from all disciplines involved in perioperative care. Further, selecting stakeholders based on the principle of reciprocity, i.e. each participant had something valuable to offer and could also benefit in return, was crucial for maintaining dedication throughout the process.

The format of consensus discussions (e.g. larger group versus small group meetings) was flexible and tailored to suit participant preferences, considering competing clinical priorities for staff. Throughout the meetings, the facilitator guided discussions through pathway components, presenting different rationales for reaching consensus and allowing clinicians to articulate their reasons for agreement or disagreement. The rationale involved a complex interplay between evidence-based guidelines, the value clinicians placed on autonomous practice, and the logistics of patient care. Through this social process of discussion, the threshold for consensus naturally emerged. For some pathway items, consensus was reached unanimously, as evidenced by explicit, verbal agreement. However, the consensus threshold could be adjusted, particularly when surgeons or anaesthetists disagreed. Compromises were made in these cases, such as allowing for ‘individual preference’ items, acknowledging that there would always be some aspects of care that clinicians may not be able to agree on.

### Consensus climate

Factors relating to the individual and the organisation were found to influence the ‘mindset’ of the consensus group driving their approach to the discussions and the overall ‘climate for consensus.’ A shared understanding of the value of pathways and demonstrating how they contribute to improved care delivery created the pre-condition for a more conducive climate for consensus-building.

Clinical standardisation was a clear goal for the organisation which drove a concerted effort to allocate adequate time and resources towards conducting consensus discussions. However, it was likely that the organisation’s approach to achieving consensus and the level of agreement needed between clinicians was influenced by how a private facility operates. Participants reported that private facilities in Australia work with “a lot of different proceduralists […] bringing their practises from their home-based organisations to here […] In some ways, every individual who comes to operate at a private hospital is the leader of their own practise” [Int.1, management]. Within this model, surgeons who practice within private facilities adhere to a set of professional norms, however, also enjoy autonomy from supervision. A philosophical question was then raised about whether private facilities have a role in standardising care or conversely, just allowing clinicians to "flex their independent clinical practice" [Int.1, management]. This may have adjusted the view of the organisation on what the consensus threshold should be and the “acceptable bound of variation” [observation, orthopaedic, 16/02/23]. Concerns over patient satisfaction levels and the financial implications or losses of reducing length of stay were also a factor raised in discussions where, compared to public institutions, ‘“public want to get people out as fast as they can’ whereas private hospitals have some incentives to keep people in for a certain period of time” [observation, nurse committee meeting, 17/11/23].

At the individual level, clinicians' mindset entering consensus discussions was influenced both by an intrinsic motivation to engage with the process and the organisation emphasising the value of implementing perioperative pathways into their workflows. For instance, surgeons were primarily driven to engage by the prospect of obtaining useful outcomes, such as patient outcomes or performance metrics, to guide their work, while nurses and allied health were motivated by the opportunity to streamline workflows and reduce the number of individual surgeon protocols they use. Although some surgeons willingly participated in the process, they became apprehensive if they perceived a threat to their autonomy and insisted on incorporating "preference items." In contrast, nursing and allied health did not appear to place the same level of importance on this autonomy and if the proposed pathways resembled suggestions rather than a standardised protocol, they questioned the purpose of participating.

### Mechanisms of consensus discussions

We found eight explanatory mechanisms which ultimately drove discussions towards consensus. Evidence-seeking formed the foundation for consensus discussions, whether from external sources like current practice guidelines, or social processes such as comparison with peer practices. Each mechanism was observed to have varying levels of relative importance assigned to them, resembling a hierarchy.

#### Evidence

The presence of strong evidence was at the forefront of decision-making, defined as having a high level of evidence in favour of and supported by one or more well-designed and well-implemented randomised-controlled studies [42]. When a topic was introduced for discussion, clinicians invariably probed, "what does the evidence indicate?" by referring to established discipline-specific clinical guidelines or current published evidence. When discussions were circling, the facilitator would use current evidence as a tool to bring the focus back to consensus and facilitate agreement. Strong evidence in favour of a particular care practice provided a strong basis for agreement and enabled swift consensus. When evidence was equivocal (or ambiguous) for a given pathway component, more experiential mechanisms were relied on to reach agreement.

#### Consequences

Potential consequences to patients or clinicians were occasionally highlighted to ensure that certain items were not excluded. This approach was particularly important when the exclusion of a practice could result in adverse outcomes. For example, even when evidence suggested that a pathway item was not needed for most patients, clinicians would consider the rare but critical situations where its absence could cause harm. Therefore, they would often agree to include the item in the pathway to mitigate potential risks.

#### ‘My practice’

Surgeons and anaesthetists would often invoke their right for individualised practice, highlighting the value they placed on autonomy of practice. Medical staff expressed a strong dislike for anything prescriptive, feeling that they had professional ownership over surgical spaces. This view was not surprising to other medical staff, rather it was acknowledged that “to get surgeons on board, there has to be ‘a bit of give and take’” [observation, spinal, 3/03/23]. Ultimately, pathways that respected this autonomy by offering ‘suggestions’ rather than rigid mandates were perceived more favourably by surgeons. At times, autonomy would be raised in the context of the responsibility doctors have to their patients. There was an underlying belief that since they are ultimately responsible for patient care at the highest level, they should have the freedom to operate based on their professional judgment.

#### Peer practice

In instances where a clinician lacked strong preferences for a pathway component, peer practices from within their discipline was the next mechanism that would promote consensus or agreement to change behaviour. For example, when clinicians were uncertain about specific patient care techniques, they turned to their peers for guidance, recognising peer practices as a credible source of evidence. When a single clinician within the same discipline exhibited varying practices, there was a tendency for them to adjust their practice to match the norms observed among their peers. However, surgeons and anaesthetists primarily looked to peers within their own disciplines for guidance. In comparison, allied health and nursing professionals, although influenced by their peers to adopt similar practices, also adhered to directives from different disciplines, even when those directives originated from outside their own areas of expertise (i.e. medical).

#### Patient preference

When evidence was lacking and clinician preferences were neutral, the theme of patient preference and what the patient may value in the pathways was raised as a consideration to reach agreement. For example, the group would consider patient’s perceptions on an item or discuss ways of reducing the burden on the patient.

#### External validation

The mechanism of external validation could overlap with the consideration of patient preferences or be activated independently. If patients were considered unlikely to have a strong preference for an item, clinicians occasionally used external influences or norms to reach consensus e.g. clinicians would defer to established practices in other hospitals. Additionally, this mechanism was present when considering the requirements of external organisations, such as accrediting bodies or registries, particularly in discussions regarding registration with external organisations.

#### Logistics of choice

Logistical and practical considerations were often raised alongside ‘evidence-based practice’, rather than serving as a standalone mechanism for consensus. Where pathway items were likely to pose challenges for staff during implementation or were unlikely to be well-received by staff, logistical concerns would be presented as a reason to agree or ‘veto’ a pathway component. Frontline staff preferences would be taken into account however, in many instances, the result would often be to use an individual surgeon preference item. Once pathways were finalised, logistical considerations became the primary mechanism for fostering agreement in developing an implementation plan on the wards. Staff deliberated on factors such as resource allocation and the timing of implementation, considering who would be responsible for delivering care and when specific pathway items would be integrated into the patient care journey.

#### Process moderator: climate of respect and collegiality

The mechanisms described were likely influenced by group dynamics where the facilitator enabled an environment conducive to open and collegial discussion. Multidisciplinary engagement proved essential to the consensus-building process and a high level of respect for each discipline’s expertise was evident in the meetings and the role they each play in patient care. However, a clear hierarchy emerged among healthcare professionals during discussions. In large meetings where medical, nursing, and allied health collaborated on pathways, surgeons and anaesthetists typically took the lead, while nursing and allied health professionals mainly contributed within the scope of their expertise or when prompted. These latter groups tended to engage more actively and freely in smaller, separate discussions. Consensus among nursing and allied health staff often revolved around 'vetoing' items within their clinical domain or when it concerned patient logistics that medical proceduralists were uncertain about.

### Hypotheses

Data analysis generated nine hypotheses (Table 3) for how context and individual determinants may influence the process of consensus. These hypotheses offer suggestions for design modifications aimed at enhancing the process of consensus discussions.

**Table 3:** Hypotheses of how context and individual variables may influence the process of consensus

***No.***	**Hypothesis**	**Description**
***1***	*The presence of strong evidence for a care item facilitates consensus and reduces conflict among clinicians due to its persuasive influence on decision-making*	This hypothesis posits that the availability of compelling evidence acts as a catalyst for consensus, streamlining the decision-making process and promoting alignment among clinicians. Strong evidence serves as a persuasive factor, minimising disagreement and fostering agreement among participants
***2***	*A) Lack of evidence for a care item activates different experiential mechanisms to achieve consensus, especially clinician preference in cases where clinicians prioritise autonomy over standardised practices*	In scenarios where evidence regarding a care practice is inconclusive or ambiguous, this hypothesis proposes that consensus becomes elusive. Clinicians, particularly surgeons, may prioritise individualised approaches over standardised practices, citing their autonomy and preference. This tendency towards individual preference items likely prolongs consensus discussions and increases the likelihood of disagreement among participants
	*B) Surgeons highly value their individual autonomy and decision-making authority, leading to a preference for pathways that offer suggestions rather than mandates*	This hypothesis posits that surgeons value the freedom to make individualised decisions based on patient needs and professional judgment. Pathways that respect this autonomy by offering suggestions rather than rigid mandates are perceived more favourably by surgeons. By allowing some items within pathways to be left to individual choice, surgeons perceive these pathways as supportive tools that enhance rather than constrain their practice
***3***	*Care items where surgeon’s perceive risk to a patient e.g. threaten patient safety or increase the burden on the patient, will lead to one of two options: 1) standardised practices by choosing the ‘easiest’ or safest option for patients or 2) surgeon preference item to ensure individualised care and ensure care is not prescriptive*	In situations where there is a perceived risk to patient safety or increase in patient burden, our hypothesis proposes two possible responses: standardisation or individualised care. Clinicians may opt for standardised approaches to ensure consistency and mitigate potential harm to patients. Alternatively, they may prioritise individualised care to accommodate patient preferences and unique circumstances, thereby maintaining a patient-centred approach while addressing safety concerns
***4***	*Face-to-face group meetings with good facilitation promote engagement in the consensus process by facilitating active participation and enhancing collective ownership over decision-making outcomes*	This hypothesis posits that direct interaction facilitates active participation, encourages open dialogue, and enhances the sense of collective ownership over decision-making outcomes. It is important to note that this dynamic can be discipline specific; surgeons and medical professions i.e. those professions with higher perceived power and high stakes in outcomes benefit from in-person meetings to engage more effectively with one another. However other professions lower in the hierarchy who may only speak when spoken to in larger meetings that include professions considered to have higher power, may speak more freely and contribute more actively when meeting with their peers in smaller, separate groups
***5***	*A) Discipline leadership can act as a social influence, shaping and guiding clinician behaviour change during consensus discussions*	When a discipline lead is engaged and actively promotes a care item that may involve a change in clinician behaviour, clinicians are more likely to agree as it can be viewed as either a form of practice validation or a leadership directive. The result of a leadership directive can be clear clinician agreement to a standardised item, or consensus by omission
	*B) Medical professionals are more likely to accept behavioural changes within clinical practices (or aspects of their behaviour) when the directive comes from medical discipline leadership or peers compared to directives from non-medical leadership sources*	This hypothesis suggests that medical professionals may be more inclined to adopt changes when they perceive the directives as coming from individuals with relevant clinical expertise and understanding of surgical practice, such as medical discipline leaders or peers. Conversely, resistance to change may be higher when directives originate from non-medical leadership sources
***6***	*Building an authorising environment where there is a focus at the institutional level on improvement builds momentum and creates sufficient change valence to initiate change*	This hypothesis suggests that establishing clear goals, demonstrating the value of pathways, and offering incentives for participation create a conducive environment for consensus-building. Institutions that prioritise quality improvement initiatives and provide incentives for pathway standardisation are more likely to garner clinician buy-in and participation in consensus groups
***7***	*Multidisciplinary representation and frontline staff involvement in consensus discussions facilitates an understanding of practice variation and promotes streamlined processes by providing insights from relevant stakeholders*	By having multidisciplinary representation in consensus groups, organisations can gain insights into practice variations and bring in multiple perspectives from stakeholders who will be using the pathways to develop flexible pathways that accommodate for diverse needs. This hypothesis also suggests that involving frontline staff in decision-making leads to more efficient processes, reduced workload, and improved alignment and respect across disciplines
***8***	*Decisions within consensus discussions are influenced by an existing and underlying hierarchical culture within the healthcare system, where tacit acknowledgment and respect for hierarchy shape the decision-making process*	The acknowledgment of hierarchy may be intertwined with the understanding of medical accountability, where individuals in higher positions within the hierarchy are presumed to bear greater responsibility for patient outcomes. Consequently, decisions put forward by surgeons may carry more weight and are less likely to be challenged. This differential power dynamic can result in allied health and nursing staff decision-making authority being restricted to veto power within their respective clinical domains. Conversely, surgeons may wield broader veto authority, even extending beyond their specific scope of practice. Moreover, discussions of practices within the surgical domain may be exempted from external input, with surgeons maintaining exclusive control over decision-making processes within this realm
***9***	*In institutions that embrace individualised approaches as part of their model, clinician autonomy becomes a pivotal factor, rendering consensus-building inherently more complex and enabling a higher threshold for ‘acceptable’ parameters of variation*	Some institutions operate under a business model that fosters a culture of individualised practice and that values and encourages clinicians to exercise their professional judgment within the parameters of safe and ethical practice. Consequently, consensus-building efforts must contend with a diverse array of clinical perspectives, preferences, and decision-making styles, reflecting the autonomy granted to clinicians in determining the most appropriate course of action for their patients. This principle may necessitate different strategies to achieve consensus compared to more standardised public settings to avoid conflict to greater variation in the pathway

## Discussion

The findings of this study highlight the importance of aligning organisational goals with individual motivations to create the optimum ‘climate’ for consensus. The Consensus Model for Standardising Healthcare also demonstrates several mechanistic pathways that drive consensus discussions, each carrying varying degrees of importance in clinicians' overall perspectives on the reasons for agreement. Overall, the core mechanisms underlying consensus discussions to develop perioperative pathways were fundamentally driven by the pursuit of evidence.

Various mechanisms held differing levels of importance in guiding clinicians' agreement and moving discussions toward consensus. Clinicians rated having strong evidence for a pathway component as most important to inform their decision-making, yet experiential evidence also emerged as key when there was no strong evidence in favour of an intervention. Numerous factors in the literature have been reported to influence clinicians’ decision-making such as evidence, patient-related or physician-related factors [43–45]. Additionally, research on cognitive biases has demonstrated how a clinician's experience and thought processes can have a large influence on decisions [46]. The fact that experiential mechanisms helped to drive discussions may reflect clinicians’ fundamental trust in the expertise and leadership within each discipline to adopt practices associated with favourable patient outcomes. For example, a study examining how dentists use evidence in practice revealed that ‘tangible’ evidence, that is evidence from their own experiences and from their peers, primarily guided their practice as this type of evidence was felt to be more concrete and therefore the most trusted [47]. It could also reflect a culture in healthcare where some clinicians such as nurses have reported feeling more confident asking colleagues or peers than they do using bibliographic databases to find specific information [48].

Identifying the case for change and establishing a dedicated 'driver' team separate from frontline clinicians in addition to consensus groups was important to drive the process. These steps may be particularly important in healthcare contexts where negotiating improvement initiatives and balancing competing priorities can be challenging [49, 50]. Interestingly, the consensus threshold was not predetermined prior to meetings. Unlike formal consensus processes that have strict criteria for when consensus is achieved [51–53], we found that the consensus threshold naturally emerged from group discussions. Studies from other fields, such as engineering, have similarly reported ‘spontaneous’ consensus and perceive group consensus as an ongoing, dynamic process where ‘communication’ is the main mechanism that leads to this ‘cognitive convergence’ [54–56]. In our study, communicating between peers to seek evidence for decisions drove consensus in developing perioperative pathways.

A naturally emerging threshold for consensus was likely influenced by each stakeholder’s presuppositions of what consensus should look like which has implications to consider. Firstly, some clinicians in our study, particularly nursing or allied health, believed that every component of a pathway should be standardised, while others thought that unless a component was evidence-based, there should be flexibility allowed in practice. This disparity aligns with a broader understanding of consensus-building, which posits that the goal is not full agreement but rather reaching a point that everyone can accept [26]. However, this difference occasionally led to conflict and raised questions about the rationale behind standardising care if elements of variation were still permitted. These conflicts could reflect work flow implications for different disciplines where for example, a small number of variations per surgeon results in a large number of options for nursing staff in recovery. This may have overarching effects on future fidelity to pathways and sustainability of implementation efforts where limited buy-in of newly implemented interventions may reduce adherence over time [57]. It is important to establish what the expected outcome and meaning of consensus is at the start of a process to ensure all clinicians are on the same page.

Secondly, if organisational hierarchy played a role in clinician’s thinking, then assumptions of which stakeholders are most crucial to obtain consensus from may affect participation. Hierarchy is deeply embedded in healthcare culture, characterised by a top-down management structure where certain professions have historically been regarded with different status levels [58, 59]. For example, nurses and allied health tend to act subordinately in deference to medical professionals [60]. One study examining the dynamics between a team of senior consultants and junior surgeons observed that decision-making within the team was influenced by both the distribution of expertise within the team, and the broader organisational hierarchy. There was an implicit understanding that the lead surgeon is the authoritative expert and would lead decision making [61]. In our study, medical staff tended to have the final say, likely due to having a higher burden of accountability. Yet, respect and collegiality among disciplines was evident and clinicians were aware of each discipline’s contribution to patient care.

The generalisability of this model to other contexts beyond perioperative care is important to consider. While the perioperative setting provided the context for developing the model, we did not observe substantial differences between surgical cohorts in terms of mechanisms and factors that influenced the process. Consensus groups included a diverse range of health professionals (e.g. anaesthetics, allied health, nursing), many of whom work across different surgical areas making the model not specific to surgeons or particular surgical cohorts. Many of the factors incorporated into the model also align with theories documented in the literature. For example, the determinants important to fostering a conducive climate for consensus closely align with other theories. Drawing from institutional theory, different institutional pressures (coercive, mimetic and normative pressures) were observed at the organisational level as a reason for engaging in a consensus process [62]. Similar to other implementation literature, alignment between both organisational goals and individual perceptions emerged as a pre-condition for engagement with the process [63, 64]. Both vertical alignment across the organisational hierarchy and horizontal unit alignment helped secure the necessary resources and structures for implementation [63, 65]. When examining the characteristics of individual healthcare professionals, the importance of medical autonomy is a recurring theme [14], suggesting that our model is applicable to settings where professionals practice with a degree of clinical autonomy and require informal consensus processes to standardise care. An example of this is primary care practices where general practitioners, who view autonomy as central to their professional identity, may need to reconcile diverse clinical approaches within a shared patient population [66, 67].

Achieving consensus around implementation processes may require greater consideration of discipline-specific issues as individual and contextual factors come into play. However, this is a widely acknowledged challenge within the implementation literature, which emphasises the importance of tailoring strategies to the specific context [68]. There are also limitations in generalising our model to formal consensus development processes, such as using the Delphi Method for clinical practice guideline development, or in situations where aspects of care are already mandated and delegated authority is in place, such as mandatory reporting requirements.

One item not included in the model and which is often contested in the literature for consensus-building is evaluating the process of reaching consensus itself. Common criteria for evaluating consensus reported in the literature are whether or not agreement was achieved or whether the agreements, in this case the pathways, were implemented. Two problems arise from these criteria. In this case, consensus was determined to be achieved in all instances and perioperative pathways were implemented. However, we are unable to determine the quality of consensus or implementation without ongoing evaluation of fidelity to the pathways and their sustainability. Therefore, ongoing evaluation may prove essential to evaluating the usefulness of this strategy and are important considerations for future research and practice.

### Limitations

This research focused on a single setting in metropolitan Sydney, which may limit the generalisability of the findings to other settings. To gain a deeper understanding of consensus across different healthcare settings, further qualitative research is needed in diverse contexts such as primary care. The sample did include a broad cross-section of healthcare professionals in both clinical and non-clinical roles within the organisations with fairly even representation from each surgical discipline. While the involvement of a small number of clinicians in interviews restricts the scope of the insights, it should be noted that our methods triangulated interview insights with those gained from observations which included many more clinicians. It was not possible to interview all targeted participants due to difficulties with recruitment. In addition, it must be acknowledged that interviewees are self-selected and may not be fully representative of their local facility.

## Conclusions

This study identified the key steps used to operationalise the implementation strategy ‘conducting local consensus discussions’ and determinants that can influence a climate for consensus. Eight mechanisms that underpin decision-making in healthcare were also identified with strong evidence at the forefront of consensus. The Consensus Model for Standardising Healthcare furthers our understanding of consensus discussions as a strategy and provides key considerations for healthcare settings looking to utilise consensus discussions.

## Supplementary Information

Supplementary Material 1. Standards for Reporting Qualitative Research (SRQR) checklist, (.pdf). Populated checklist of ‘Standards for Reporting Qualitative Research’ reporting guidelines indicating how the manuscript adheres to the relevant guidelines.

Supplementary Material 2. Topic guide for semi structured interviews utilised for surgeons, (.pdf). Provides the topic guide initially piloted by the research team and used to guide the interviewer in the semi-structured interviews for patients.

Supplementary Material 3. Topic guide for semi structured interviews utilised for allied health and nursing staff, (.pdf). Provides the topic guide initially piloted by the research team and used to guide the interviewer in the semi-structured interviews for clinicians.

Supplementary Material 4. Topic guide for semi structured interviews utilised for leadership or management staff, (.pdf). Provides the topic guide initially piloted by the research team and used to guide the interviewer in the semi-structured interviews for clinicians.

Supplementary Material 5. Initial Codes, Categories, and Integration into the Model, (.pdf). Display of initial inductive codes and categories derived from the data and examples of theoretical codes and inductive codes were incorporated and fit into the model along with some mechanistic pathways emerging from the inductive codes.

Supplementary Material 6. Supporting quotations for determinants, mechanisms and processes of consensus, (.pdf). Verbatim quotations from interview transcripts and segments from observation field notes are presented in support of the determinants, mechanisms and processes of consensus.

## Data Availability

The datasets used and/or analysed during the current study are available from the corresponding author on reasonable request.

## Abbreviation

SRQR: Standards for Reporting Qualitative Research
