# Supplementary material for: A qualitative study of how clinicians reach agreement in perioperative pathway development: the Consensus Model for Standardising Healthcare
Source: Implement Sci Commun. 2025 Feb 4;6:17. doi: 10.1186/s43058-025-00699-9 (PMC11796167; doi:10.1186/s43058-025-00699-9)
Supplement: Supplementary file 3 — Supplementary Material 3. Topic guide for semi structured interviews utilised for allied health and nursing staff, (.pdf). Provides the topic guide initially piloted by the research team and used to guide the interviewer in the semi-structured interviews for clinicians. [file 43058_2025_699_MOESM3_ESM.docx]

**Topic Guide: Nursing/Allied health interviews**

| **Title** | Implementation of evidence and consensus-based perioperative care pathways |
| --- | --- |

The semi-structured interviews will be conducted retrospectively for key clinical and non-clinical hospital staff who were previously involved in the development of perioperative pathways for the elective hip and knee replacement surgical cohorts; and prospectively for staff currently involved in the development and implementation of new pathways. The aims of the interview are to:

- Determine the experiences of staff involved in implementing standardised peri-operative pathways.
- Explore the stakeholder knowledge of the care pathways within the hospital setting.
- Assess the integrity, fidelity to and feasibility of the intervention.
- To understand the key barriers and facilitators to implementation of standardised peri-operative pathways from the perspective of both clinical and non-clinical hospital staff.
- To synthesize data from both patient groups to gain a broader understanding of how standardised care pathways are implemented and the impact of these pathways.

**QUESTIONS:**

1. Can you please introduce yourself and explain your role?
2. How long have you worked in healthcare?
3. How many of those have been at [Name of] Hospital?
4. What was your experience/what has been your experience implementing the [SURGICAL COHORT] peri-operative pathway/s?
5. How familiar are you with the particulars of the clinical pathway/s? Can you talk through some of the changes from previous practice?

- What worked?
- What didn’t work?

1. What are your views on the standardised clinical pathways that were implemented/are being implemented?

- Strengths
- Weaknesses

1. Did you and other clinicians tend to follow the new processes/procedures?

- Why do think that is?

1. What challenges, if any, did you encounter in implementing the clinical pathways?

Prompts;

- Workload and time
- Staffing
- Personal preference/views/autonomy
- Inter-professional collaboration
- Other

1. What was important to facilitating the implementation of the clinical pathways?

Prompts;

- Organisation
- Resources
- Staffing/Inter-professional collaboration
- Support/Monitoring of fidelity/quality
- Other

1. Were there any other lessons learned about implementing standardised clinical pathways?

*Additional probing question if not covered in answer above:*

1. What are your suggestions to improve future implementation of standardised clinical pathways for [SURGICAL COHORT] surgery?
2. What do you see as the effects and value of implementing standardised clinical pathways for [SURGICAL COHORT] surgery?
3. Overall, how feasible is/was it to implement standardised clinical pathways for [SURGICAL COHORT] surgery at [Organisation] or private hospital settings more generally?
4. Is there anything else you would like to add that has not been covered here?
