# Supplementary material for: A qualitative study of how clinicians reach agreement in perioperative pathway development: the Consensus Model for Standardising Healthcare
Source: Implement Sci Commun. 2025 Feb 4;6:17. doi: 10.1186/s43058-025-00699-9 (PMC11796167; doi:10.1186/s43058-025-00699-9)
Supplement: Supplementary file 5 — Supplementary Material 5. Initial Codes, Categories, and Integration into the Model, (.pdf). Display of initial inductive codes and categories derived from the data and examples of theoretical codes and inductive codes were incorporated and fit into the model along with some mechanistic pathways emerging from the inductive codes. [file 43058_2025_699_MOESM5_ESM.pdf]

Inductive coding

Codes

Initial inductive codes grouped	Other comments	Focussed coding	Concepts
Desire to improve<br>Outcomes as an incentive<br>Reason for following pathway<br>Measurements of pathway success<br>Goal directed<br>Focus on improvement<br>Institutional focus on improvement<br>Goals of hospital or organisation<br>Measures of success for buy-in<br>Setting the goals and foundations<br>Understanding of need for pathway		Goal oriented<br>Individuals and institutions focus on improvement<br>Using outcomes as an incentive	Case for change and can see the value of the pathways
Drivers or motivation to engage<br>Responsibility to be involved<br>Engagement with meeting<br>Need Buy in<br>Clinician engagement and awareness<br>Proactive and engaged clinicians<br>Professional responsibility<br>Getting everyone on board	What are the different drivers or motivators for clinicians or others to buy into the process and engage with the process of consensus?<br>Is there a responsibility for the clinician to be involved in this process and therefore engage with it?	Engagement needed by clinicians and staff<br>Need buy in<br>Proactive and engaged clinicians as a facilitator	

Difficult to get buy-in<br>Individuals can see value in pathways	Where (or whom with) does the responsibility lie?<br>Awareness of pathways and making sure there are methods to engage clinicians as part of the implementation plan		
Value of clinical pathways<br>Consensus process encourages change<br>Different perceptions of main value<br>Provides useful outcomes and data	Going through the consensus process itself can actually encourage change. It is not just about reaching the end goal of having a pathway developed.<br>Is this related to what is going to impact their specific discipline overall?<br>Useful for hospital to provide measures of performance and success.	Value of clinical pathways<br>Value of the consensus process	Value of pathways
Streamlined processes built into the system<br>Support to implement<br>Workarounds<br>Easy for new grads or after hours staff to follow<br>Training staff<br>Streamline processes<br>Dedicated time to complete tasks related to pathways<br>Incentives or reimbursement provided		What's important to staff?<br>How can we help them?<br>Improved staff workflow<br>Training and education	
Involvement of different disciplines<br>Anaesthetists	This meant a limited understanding of clinical work and also how the processes at the hospital work.	Consensus group multidisciplinary<br>Alignment between different groups and clinicians<br>Brings in lots of different perspectives	Alignment of views between all involved

Acknowledgement of other disciplines<br>Use of feedback from staff<br>Consultants not included in meetings<br>Lack of MDT involvement<br>Project manager useful to drive project<br>Surgeons need to adopt the pathways<br>Disconnect between leadership and clinical staff<br>Roles of members<br>Coordination and frontline staff engagement<br>Inclusion of other members e.g. education, Q + S<br>Still need more frontline clinician involvement<br>Examples of staff involvement<br>Implementation champion not internal or a clinician<br>Need MDT involvement in initial phases<br>Teamwork<br>Use of implementation taskforce<br>Use of smaller groups<br>Implementation champion that knows the setting<br>MDT involvement<br>Collaboration<br>Doctors need to be involved	

Assistance from researchers<br>MDT collaboration<br>Felt valued and included<br>Minimal bedside involvement<br>Value input from staff<br>Facilitator role<br>Implementation champion		
Professional experience<br>Trust<br>Discipline leads and strong leadership needed<br>Directive from senior as a cause for change<br>Lead by discipline lead<br>Power over other staff	Strong leadership needed<br>Listen and follow a lead<br>Value of leadership to drive projects and change behaviour	Value of leadership to drive projects
Achieving consensus in public versus private hospitals<br>Business model and considerations<br>Governance structures in public hospitals<br>No consequences in private hospitals<br>Funding structures as a barrier<br>Financial gains for having increased LOS<br>Cost<br>More cases seen<br>Variation inherent within model or hospital set up<br>Private hospitals allow autonomy v Public	Public versus private hospital business model<br>Allow for more variation in practice<br>Finance and governance means more variation	Institution type can impact consensus process

Enables variation by allowing for individual ways of practice<br>Reduces costs			
Flexibility and tailored processes to each discipline<br>Need some degree of flexibility to sustain<br>Flexibility of other staff<br>Different group approaches to consensus<br>Fractions within each discipline<br>Differing patient processes for different surgical disciplines	Admin or implementation champions being flexible to meet with clinical staff when able.	Acknowledging differences in each discipline	Climate of respect and collegiality
Agreement<br>Group mentality<br>Appeasing others<br>Collegiality<br>Opening up the floor to everyone<br>Delegate tasks<br>Give and take<br>Teamwork<br>Flexible pathway<br>Already practice in that way	Pre-existing relationships between colleagues.<br>Modifications need to be able to be made to the pathways.<br>What is everyone else doing?<br>Not much change required at the clinician level as many already practice in that way	Group mentality<br>Group agreement<br>Collegiality<br>Working well together towards a common goal? OR different ways of working together	
Acknowledgement of other disciplines<br>Expertise<br>Other clinicians practise<br>Defer to others with more knowledge<br>Teamwork<br>Collegiality<br>Staff training opportunities	Pre-existing relationships between colleagues<br>Rather narrow view<br>Using people's specific expertise	Value other people’s input<br>Seeking other’s expertise<br>Collaboration, want everyone to succeed	

Conflict<br>Differing opinions<br>Dominant figures<br>Hierarchy of decision making<br>Profession superiority<br>Professional confidence<br>Individual practice<br>Strong communication<br>Undermining<br>Competitive<br>Individual opinion<br>Want to voice their opinion<br>Individual views of clinicians may differ<br>Presented as a choice to change or follow the pathways<br>Individual knowledge and skills<br>Individual drive to further project<br>Individual agenda or suggestions based on one's agenda<br>Individual harm versus harm to other areas	Drive of the individual (clinician or other involved in the group) to continue to move the project along.	Individual agendas and methods<br>Individual driving opinions	Value of professional opinions and autonomy, value of autonomy and individualised practice
Always areas where consensus can't be achieved<br>Is a suggestion not a pathway<br>Perceptions of the pathways<br>PRN items<br>Where do you draw the line	Some variation in practice is ok but where then do you stop if you accommodate for everything?	Difficulty in achieving unanimous consensus<br>Flexibility in ideas of consensus needed (linked to individual ways of practising)	
Perception that clinicians need or want to have their say	Use of surgeon practice as a means of deciding before bringing in other reasons	Individual practice<br>Clinician Ownership of spaces and dislike prescriptive nature	

Clinical challenges with differing practices<br>Are doctors doing to change<br>Ownership of certain spaces<br>(This is their space and someone outside that space can't make decisions for me)<br>Influence of hospital on decision making<br>Other health disciplines versus doctors<br>Perception surgeons not aware<br>Differing clinician practises<br>Discipline perspectives<br>Preference versus evidence based<br>Surgeon practise over other reasons<br>Value autonomy<br>Prescriptive<br>Surgeon perceptions of practise<br>Surgeons may not do as they're told<br>Prior or current experience or practice	Can't be too prescriptive to other doctors<br>Idea that surgeons may not be aware of what's going on and that they don't really engage in the processes<br>Draws on own experiences and values cognitive-psychological approach	Will doctors change behaviour?<br>Doctors versus other<br><br>Value autonomy	
Acknowledgement of time, effort or other<br>Highlighting issues with current practices or logistics<br>Other responsibilities or timing<br>Responsibilities to patient<br>Staffing<br>Raising different or new issues	Clinical load can impact on attendance/involvement<br>May include other responsibilities	Clinical duties come first<br>Importance of other responsibilities value clinicians' time<br>We don't want it to cause extra workload.<br>Institutions need to prioritise consensus discussions	Allocating time and resources to consensus discussions

Involves extra workload<br>Clinical responsibilities<br>Time burden on clinicians			
Coding, tracking KPIs, technology incapable<br>Comparison to other institutions<br>External requirements<br>Reputation of the institution or other<br>Focus on practice change<br>Minimal changes required assumed<br>Variation hasn't been an issue in the past		Value placed on reputation<br>Must be same as peers<br>What are other’s doing? What can we learn or do better?<br>We care what others think?	Priorities of the institution
Patient centred care<br>Early discharge<br>Staff or patient education needed<br>Improved patient outcomes or care<br>Patient education<br>More cases seen<br>Patient care<br>Rehabilitation<br>Patient education<br>Affects patient care		What’s in it for the patients?<br>How can we improve patient care?<br>Improved patient outcomes<br>What do patients get out of this?	Patient centred care
Evidence based practice<br>Variation in practice important to developing pathways<br>Equivocal evidence<br>Improved practice or variation	Drawing on the evidence to make the decision	Focus on EBP<br>What does the evidence say?<br>Improved practice according to EBP	Seeking evidence and evidence-based practice should guide care

Promotes EBP and adherence<br>Reduced post-op complications			
Implementation plan<br>Technology resources<br>Implementation successful<br>Adherence measures<br>Compliance<br>Learnings from initial pilot work<br>Governance<br>How to ensure patient meets the pathway<br>Importance of formalising pathways<br>Audits for feedback purposes<br>Documentation<br>Need for resources and education in imp plan<br>Staffing<br>Technology issues affecting implementation<br>Support from hospital	Issues with documentation may be that people are not documenting correctly which will make it difficult to examine adherence and keep track of KPIs/fidelity.<br>Methods of operationalising the pathways, ensuring all clinicians are aware of the pathways, establishing awareness and buy-in.<br>Codes and ideas relating to initial pilot work.<br>Need to measure adherence to the pathways as an outcome.	Governance structured needed<br>How do we ensure we meet the pathway<br>Will there be consequences<br>What are the outcomes<br>Implementation plan needed	
Extra workload<br>Complex patients<br>System level issues<br>Available resources to commit<br>Patient barriers<br>Processes for rural patients<br>Medical processes stopping patient meeting pathway<br>Comorbidities and other factors<br>Technology slow progressing<br>Logistical issues	Limited resources previously available to commit to developing clinical pathways.<br>Affect mobility to achieve the pathways.<br>For example, unable to upload things to Trackcare<br>Issues with the system itself, could also relate to institutional theory	System level issues affect consensus<br>Patient issues affect consensus<br>Logistical and resource issues affect consensus	Logistics of choice

Difficulty with patient selection for pathways<br>Patient perceptions or understanding<br>Implementation barriers<br>Logistics of pathways<br>Flow on effects impacting fidelity	Flow on or logistical or clinical issues that affect a clinician's ability to adhere to aspects of a pathway		
Promoting change at the individual level<br>Resistance to change<br>Open to changing one's own practice<br>Cultural change difficult<br>Slow to see improvements and change<br>Perception medical will comply	Clinicians have a responsibility to their patients and they feel that they know what's best for their patients and so should have freedom to exercise how they want to practise.<br>Improvements/rewards/benefits not immediately seen<br>Closely relates to teamwork and some themes overlap	Need change at the individual level<br>Open to changing practice	Behaviour change and motivation to engage
Behaviour change<br>Behaviour change<br>Audit and feedback causes behaviour change<br>Nursing or Allied health open to change<br>No conversations about translation	Not enough conversation about how to translate these into practice or how this will work. Not enough discussion from other staff, seems to stop at those meetings<br><br>Surgeon has a usual way of practice but even when they may want something different, clinicians may deliver care in that usual way due to an assumption that's the way it should be	Behaviour change at the individual level needed<br>Different methods can promote behaviour change	

Assumptions based on individual surgeon practice			
Staff capabilities or hospital culture factors<br>Cameras off<br>Challenges with meeting online<br>No response to emails<br>Acceptance by omission<br>Staff unprepared for meetings	Assume consensus has been achieved when people are given the choice to respond/be involved but then haven't responded.	Disengaged with process	

Bringing back to the holistic focus<br>Defer to others with more knowledge<br>Highlight controversial issue<br>Highlighting agreement<br>Highlighting issues with current practice or logistics<br>Individual apologises for their view<br>Individual ways of understanding and learning<br>Modifying behaviour when being observed<br>Moving on when solution not present<br>Opening up the floor to everyone<br>Placating<br>Strong communication<br>Terminology specific to professions<br>Undermining<br>Voicing opinions	Communication within meeting,<br>Rather narrow view<br>Processes need to cater to the different needs of people involved in the consensus process<br>People agree but they still need to have their say and voice their opinions	Communication styles used in meeting	Climate of respect and collegiality
Prior or current experience<br>Implementation champion who knows context<br>Terminology specific to profession<br>Implementation Processes and Perceptions<br>Consequences		Clear –<br>They understand what we mean and need<br>they are like us<br>Easier if everyone understands and has knowledge across the process	

Sitting face to face or close contact<br>Meeting online<br>**Time constraints**<br>Communication and follow-up with people involved<br>Environmental influences<br>Group dynamics	Context of the meeting and any potential impacts of these contextual factors<br>Ensuring that a facilitator or another champion makes sure there is regular communication and is able to keep everyone involved, engaged and informed on progress.<br><br>How does the group work? What actions do people take? How do people behave in the group to reach consensus?	Contextual factors can impact on processes<br>Role of context in shaping consensus processes	Impact of the consensus context
Other methods to achieve consensus			
Agreement as current knowledge	Agreement as appears to be current knowledge and clinicians aware it is best practice but it may not be done for different reasons at the hospital		
Consequences	Fear of getting penalised or fear of consequences. What happens to the individual if you don't adhere?		
Incentives			

Examples of theoretical and inductive codes into model and mechanisms:

Key:

Red font: represents how inductive initial codes fit into mechanisms

Highlighted font: represents some of the theoretical codes incorporated into the model

Outcome: ‘Consensus’ achieved - underpinned by the process of seeking evidence

Different consensus types

Different approaches to consensus create different meanings of consensus

- Unanimity
- acceptance by omission – no one says anything so assume that they agree
- discipline/discipline lead says yes on everyone’s behalf – facilitated
- Individual ideas (Value of professional opinions and autonomy, value of autonomy and individualised practice**)**

Different ways of agreeing/getting to this point when nutting out the specifics of the pathways

- What do the guidelines/published evidence say?
- EBP → strong evidence for a topic → standardised item
- Equivocal evidence → surgeon practice over other reasons, dislike prescriptive → PRN
- What are the consequences to the individual or the clinician by the choice made?
- ‘My practice’ is important
- Responsibilities to patient (patient-centred care, affects patient care)
- Individual ways of practising (Value autonomy) → discipline lead issues directive → consensus/acceptance by omission

- Value autonomy → individualised (PRN) item
- Private insititution allows for more variation (business model and needs surgeons) → Facilitator opens floor to everyone → Surgeons value autonomy and individual practice OR feel responsibility for patient so have own preferences → Expressing opinions and each have a right to their own preference →conflict to agreeing → presented as a choice to change → surgeon practice over other reasons (this is how I practice) →PRN items → uncertainty amongst other staff and will people follow pathways / question are doctors going to change
- What is best for the patient and what will they need? Patient-centred focus
- Expressing opinions and each have a right to their own preference →Discussion of poor patient outcome or safety or responsibility to the patient or burden on patient→either 1)standardise and chose option easiest for patient or 2) 'as per surgeon or as per patient' to ensure individualised care and make sure not prescriptive
- What does the most senior or experienced people/person say should be done? Professional expertise, directive from leader/DL, I'll agree if the rest of the group does (group mentality)
- What are other institutions doing and if we're not doing it, then should we?
- Logistics of the choice made - how could this work in practice logistically?

The process of 'making it happen' - there are different ways of conducting consensus discussions:

- Individuals versus teams – collegiality and culture
- Larger groups in person for discussion +/- break off into smaller groups to discuss more specifics of the pathways
- Facilitator opens up the floor to everyone? → Surgeons want to voice their opinion → differing opinions or differing clinician practices → conflict → role of facilitator (highlights agreement, bring back to holistic focus/give and take) → move on when solution not present OR consensus OR PRN item
- Differing opinions/practices → role of facilitator (give and take, direct conversations etc.) OR meeting attendees 'appeasing others' or 'placating' → collegiality → agreement
- Differing opinions/practices → role of facilitator (give and take, direct conversations etc.) OR meeting attendees 'appeasing others' or 'placating' → dominant figures → decision making hierarchy/medical accountability →AH/Nursing veto only
- Discipline lead/experienced clinician working with facilitator
- Discipline lead does work → viewed as a directive → leadership causes behaviour change → acceptance and agree
- less engagement with consensus/no response to emails → unclear consensus/acceptance by omission
- Discussing online via zoom or via email – competing responsibilities → less engagement with consensus/no response to emails → unclear consensus/acceptance by omission

Consensus and implementation group established:

- Discipline leadership
- Facilitator
- Implementation taskforce/steering committee
- Clinicians (Step 1: smaller group to decide and then Step 2: out to the rest of the discipline to agree)
- Fractions within each discipline
- Surgeons involved (no anaesthetics or frontline staff involvement) → understanding variation in practice amongst disciplines → relevant stakeholders have their say → streamlined processes or flexible pathways or less workload (versus opposite)

Group established by:

- Leadership saying who should be involved
- Starting with doctors and they say who else should be involved
- Starting with nursing and then they suggest which doctors should be involved
- Institutional focus on improvement → Set goals and foundations → explain/understand needs and value of pathways AND incentives for pathways (measures of success/provides useful data) → establish buy-in → involvement in consensus group (all clinicians) and more inclined to agree to standradise more things (nursing/AH moreso)

Consensus 'climate' and context important in facilitating consensus

- Differences in achieving consensus in public versus private institutions
    - financial goals and factors impede processes
    - variation inherent within private model
    - private hospitals allow for more autonomy
- Common goals facilitate consensus - aligning service provision priorities (other clinical responsibilities compete/impede/delay the process of achieving consensus
- Different meaning and value placed on outcomes of discussions - Clinicians have different priorities and reasons for buy-in
- Focus on improvement/ Setting goals and foundations
- Institutional focus on improvement → Set goals and foundations → understanding of the needs and VALUE of the pathways→goals of institution align with clinicians (own desire to improve/individual desire to further project) → involvement in consensus group (all clinicians)
- Funding structures
- Clinician/individual 'climate'
- Open to changing practice → buy in → behaviour change (audit and feedback can assist for ALL, should be individualised for surgeons to cause BC)
- Audits and data to achieve buy-in successful (this is where we need to change)

- Perception that some surgeons not open to changing practice/may not do ‘as told’ → perception that there are always areas where consensus can’t be achieved → present as choice to follow pathways → surgeon preference items
