# Supplementary material for: A qualitative study of how clinicians reach agreement in perioperative pathway development: the Consensus Model for Standardising Healthcare
Source: Implement Sci Commun. 2025 Feb 4;6:17. doi: 10.1186/s43058-025-00699-9 (PMC11796167; doi:10.1186/s43058-025-00699-9)
Supplement: Supplementary file 6 — Supplementary Material 6. Supporting quotations for determinants, mechanisms and processes of consensus, (.pdf). Verbatim quotations from interview transcripts and segments from observation field notes are presented in support of the determinants, mechanisms and processes of consensus. [file 43058_2025_699_MOESM6_ESM.docx]

**Additional File 6. Supporting quotations for processes, mechanisms and determinants of consensus**

Verbatim quotations from interview transcripts and segments from observation field notes are presented in support of the processes, mechanisms and determinants of consensus. All quotations and observation segments are de-identified, and pseudonyms are used where applicable.

| **Consensus concept** | **Supporting quotation** |
| --- | --- |
| ***Processes*** |  |
| ***Case for change identified*** | “I trained in Florida and America and also in England and found that, you know, there were established programmes on enhanced recovery protocols. And then I came and compared it to what we're doing here and realised that we're not even close. We weren't even close to what you know, that everybody had individual practice and then a lot of improvements can be made in length of stays by changing the way we do things.” [Int 5, clinician] |
|  | “The data that we had available to us a few years ago suggested that we did have issues with, potential issues, with increased length of stay compared to peer cohorts. Other issues around perhaps increased utilisation of intensive care units, as with the Joint replacements, high utilisation of inpatient admitted rehabilitation.” [Int 1, management] |
|  | “For a lot of our clinical cohorts, we have essentially 1 clinical guideline per practitioner of that particular surgery surgery or procedure. So let's say in joint replacement it's one of the egregious examples, we have about 15 surgeons who are doing joint replacement surgery and we had 15 clinical guidelines which showed greater or lesser kind of concordance with one another […] I think that's probably the remit of an academic Health Sciences Centre, is that you should be able to say, we look after people with with best current up-to-date evidence-based practise rather than just what the particular practitioner of the day happens to bring from.” [Int 9, management] |
| ***Driver team appointed*** | “what was helpful, I think is having someone in the project and steering that, but at the same time that person isn't a clinician working there, so they may or may not have a full understanding, you know, a full understanding of how the service works […] or have a clinician that actually is a service to lead the development of it I think is better like I think currently. So for example [facilitator name] yeah, being involved, he's been here for a long time. He has worked on the wards you guys have. He has a good understanding and so therefore I think that really is better because he understands probably the nuances of the differences and understands the demands on the ward” [Int 4, clinician] |
|  | “So for me where I sit in the organisation, I feel there's been appropriate consultation […] However, perhaps not enough at that […] that patient facing level […] So people who are going to be the ones who are going to implement the pathways and follow these pathways and and give us the information that we need around, um outcomes of the pathways, I feel like there's a little link missing there.” [Int 8, clinician] |
| ***Consensus group established*** | “Harry then prompts for who else should be involved in this group aside from nursing and medicine.” [observation, cardiac, 6/03/24.] |
|  | “I think we're getting a broader representation of, I think none of the meetings before had an anaesthetist in it. And from a physio perspective we felt that there needed to be [… ] Because some of our barriers were. Why were we not getting patients up day zero. PCA's were getting in. The way there were no, you know drip poles and things like that, and patients were fainting cause they had spinal morphine. And so physios were scared to get patients up because they were vasovagal […] So, so, you know, there was no avenue to voice our concerns and discuss that. So in the initial group, whereas now I think they're, yeah, having more representation and there's more opportunities to discuss those sorts of things.” [Int 4, clinician] |
|  | “This just started with the premise that each of these pathways we would have a defined consensus group […] It would be the lead of the discipline, or if not actual discipline lead like one of every close delegates. And then a cohort bigger or smaller of engaged clinicians who worked within that discipline, who had, let’s just say an interest in research evidence and evidence in an interest in improving clinical practise through standardisation and who would be present enough to help to make decisions and respond and turn things through and as well as having then that sort of core group of medical practitioners, then you would have sort of lead representatives of the other stakeholders in both developing and implementing the thing. So, you might have like a a senior nurse or a nurse and or a nurse educator, a senior allied health practitioner, which in the joint of placements obviously would be a physiotherapist, um, depending on the particular group and what parts of the hospital the patient cohort went through, your models have representatives dip in or out of the consensus group from things like preadmission or anaesthetics or perioperative services.” [Int 9, management] |
| ***Determine format of meetings*** | “We've, getting people together is very, very difficult because they're also busy. So we did it on zoom teams.” [Int 3, management] |
|  | John suggests that “we work backwards” by having nursing staff work with Harry first to see what aspects of post-op care may need to be standardised first which can then be brought to the surgeons for discussion. [observation, breast cancer, 21/03/24] |
| ***Preparation for meetings led by facilitator*** | Harry also consulted the infection prevention nurse about follow-up in PAC (individual memo - this emphasises the multifaceted nature of making a pathway and how many people Harry has had to consult to develop pathways on a multitude of issues). [observation, spinal, 17/08/23] |
|  | Harry then shows an exemplar of a pathway (orthopaedic document) that has been created from this work to demonstrate how it could look, what may be included. [observation, breast cancer, 21/03/24] |
| ***Rationale for agreement introduced*** | A Cochrane review is brought up regarding ice therapy. Michael says that ice is important but Harry refers back to the evidence that there is no effect. Bruno agrees with Harry as ice machines can be cumbersome […] Michael states that they should be able to provide ice if they want to as it isn’t harmful. They begin to discuss autonomy and the issues that you can’t mandate everything. Bruno raises the point that you’ll find it difficult to mandate everything as everybody has their own experiences “so you have to let them do what they want.” [observation, orthopaedic, 16/02/23] |
|  | Harry then brings the room back to the point of this process, to achieve consensus-based guidelines that recommend based on strong evidence. There has to be room for ‘flair and individual approaches’ but the idea is to “make these processes more streamlined not to impose process but to reduce unwarranted variation.” [observation, orthopaedic, 16/02/23] |
|  | Another point that Harry seeks Curtis’ input on is the logistics and processes of referring patients to PAC. Curtis says that in the microdiscectomy/single level decompression context, PAC is often not warranted. He also reports that surgeons should and would have their own arrangements for patients that aren’t straightforward, not necessarily going straight for PAC. [observation, spinal, 17/08/23] |
| ***Refine pathways based on feedback*** | Curtis feels that a more general approach would be best where a general email is sent out to the wider discipline saying “this is what we’re planning to implement as a clinical guideline…let us know your comments which will be considered for ongoing iterations…” Curtis is keen to include the wording that “this is a working document” as that stops people “getting upset about things that they don’t agree with.” [observation, spinal, 17/08/23] |
|  | “And it's going kind of this process of iteration and reiteration and now let's ask these questions and can we get you know, consensus on these things or what do we think there might be a variation and then that variation.” [Int 9, management] |
| ***Mechanisms*** |  |
| ***Evidence*** | Harry also says that it’s helpful if there are published guidelines available that can be used to draw evidence on as when people don’t agree with a point, then you can lean on the fact that it is included in a published guideline. They all nod along. [observation, breast cancer, 21/03/24] |
|  | A conversation regarding alignment with evidence-based practice and guidelines ensues and no one seems to have a problem or issue with adhering to evidence-based guidelines. The mention of evidence seems to quieten the room most of time. Victor at one point even says “I think you’d be pretty brave to go against the CEC guidelines.” [observation, orthopaedic, 30/03/23] |
|  | “Other areas like VTE prophylaxis, where where, um. Again, the evidence may not be, ah, equivocal. And you'll find surgeons will be influenced by particular peers that they worked with or their previous experience. Or you know that patient that they had then that they did this with and that didn't work because they had this bleed or so on. And then if the, if the evidence isn't so completely robust, people are always gonna kind of be guided in that by by what their personal experience has been. And then can you influence that? Well, you probably can't say you've gotta do a if there isn't a published paper that says, you know, we've run 10,000 people through this trial and we know that if you do process A one person gets a DVT and if you do process C, 10 people get a DVT yeah. You know, if there isn't that degree of evidence, then is it, It's hard to actually put in a guideline, ‘well, you have to take that particular path.’” [Int 1, management] |
| ***Consequences*** | Michael continues and appears to try to justify his question with context and history discussing that if a surgeon doesn’t “tick that box” then you get penalised so it’s easier just to tick it to prep the patient for admission to the intensive care unit. Victor is quick to shut down that comment using a clear and concise statement that contradicts Michael’s statement. He says very few patients end up in ICU which seems to imply that Michael’s concern is unwarranted. [observation, orthopaedic, 16/02/23] |
|  | There appears to be differing perspectives on what the important things are here. Michael says if we have no O- blood and a patient needs it then “we have no leg to stand on.” [observation, orthopaedic, 16/02/23] |
|  | “Really because we're a single institution that's a little bit isolated from other institutions, even though the evidence suggests that, generally speaking group and holds don't need to be done, there are particular things about our particular organisation that means in that rare happenstance where something goes wrong it it's it's perilous. So we agree that the evidence suggests that probably a group and hold aren't routinely used, but we're going to interpret that through the lens of what are the negative consequences [...]in that rare circumstance, if we hadn't done it. So we're gonna put it in the pathway, even though we recognise that there's evidence to suggest that for the majority of patients it’s not needed. [Int. 9, management] |
| ***‘My’ practice*** | As the discussion becomes more to “mandating” or prescribing certain practices where there may be more variation, the surgeons bring up their autonomy or other practitioner’s autonomy. Bruno asks “I have a question…we are telling people’s anaesthetists to write something and will they write what you tell them to write? They have preferences. These need to be recommendations.” Victor agrees to this wording change and that these pathways should be a guideline rather than a mandate. Harry agrees to change this language. [observation, orthopaedic, 30/03/2] |
|  | “That actually comes under the anaesthetists and not the surgeon, and therefore you need a consensus from the anaesthetists just to say I want to streamline, you know, can we streamline the approach on that? Again, there's another group of ministers who see it differently. ‘I do it my way’. So it's an uphill battle. [Int 3, management] |
|  | As the meeting wraps up and Harry explains the next steps in the process of consensus and how they are going to approach achieving consensus, Curtis makes an interesting comment on “the fact that surgeons have gotten so precious about their own rights based on their experience.” He indicates that this could be a problem in the path to establishing consensus.’ [observation, spinal, 30/03/23] |
|  | Enzo agrees and says that buy-in from “lots of intensivists” will be required for success as one surgeon may be more agreeable to change, but “if I have a different intensivist then that patient is staying in ICU for longer.” [observation, cardiac, 6/03/24] |
|  | He states “I’m loathe to have lots of articles in a consensus pathway that says individual preference but it looks like this is another one.” This idea does not seem to bother the clinicians. Michael states that they should be able to provide ice if they want to as it isn’t harmful. They begin to discuss autonomy and the issues that you can’t mandate everything. Bruno raises the point that you’ll find it difficult to mandate everything as everybody has their own experiences “so you have to let them do what they want.” The decision is made to leave the ice article as ‘per individual surgeon.’ [observation, orthopaedic, 16/02/23] |
|  | Maureen suggests to give the surgeons a choice of five options to choose from for example. The surgeons are ok with this, promoting for some type of flexibility. Michael says that you should likely ask each surgeon what their preference is to see if you can get a smaller number of choices to choose from and if there’s an outlier then you may just have to accommodate for that outlier. [observation, orthopaedic, 30/03/23] |
| ***Responsibility to patients*** | “When the slide about ‘day of surgery admission’ is shown and Harry mentions that there is one outlier, Nancy, Trish and Claire all say “how do we get them to change?” Harry provides some examples of how this could be approached with a focus on assuaging the concerns that day of surgery admission is not going to lead to “lesser care.” [observation, implementation meeting, 10/10/23] |
|  | An additional reason that is brought to the table for surgeons being against mandating goes beyond autonomy to patient care. For example, Michael discussed how patients react differently to different drugs and that some people have side effects to non-steroidal anti-inflammatory drugs. Emily agrees saying “that’s the other concern.” [observation, orthopaedic, 30/03/23] |
| ***Peer practice*** | He raises it and it appears that Bruno is the only one still completing that action in practice whereas nobody else is. Bruno, in an agreeable manner, asks what everyone else is doing and when he learns that he is the only one he states, “easy, strike off” indicating that he is happy to change his practice. [observation, orthopaedic, 16/02/23] |
|  | John asks Ginny what her protocol is and if she uses heparin. Ginny says she uses Clexane. John then asks when she uses 40mg vs 20mg to which Ginny responds with her current practice. Ginny then says, “I’m happy to go with the consensus.” [observation, breast, 9/05/24] |
| ***Patient preference*** | Harry further probes on this point about PAC for ‘younger people’ and if this usually occurs. Curtis raises the point that, according to current evidence, there is no additional value added to patient care by sending people to PAC for routine tests e.g. pathology/bloods, chest Xray's etc and that usually, all that results from these tests are incidental findings. Harry agrees with this as Curtis discusses the extra burden and problems that these incidental findings can raise, and that people will often spend a lot of time then chasing up these results. [observation, spinal, 17/08/23] |
|  | The topic of joint school is brought up and Harry expresses that even though it is non-evidenced based, patients do like it so the question remains of should this stay in the pathway. Michael steps in before anyone else saying that when institutions have applied it, he has gotten feedback from patients that they love it as it gives them feedback and insights so it should be kept. [observation, orthopaedic, 16/02/23] |
| ***External validation*** | On a couple of other occasions, Harry states “I’m not sure about the evidence on this”, wanting to be guided by evidence-based practice and Curtis then brings in common practice at other hospitals e.g. public hospitals. [observation, spinal, 30/03/23] |
|  | Harry asks the question when moving onto the next item – “do we want to commit resources to doing 6-month follow-up measures and linking to the AOOS registry? Bruno mentions that if we want to be serious teaching hospital then we should commit. [observation, orthopaedic, 30/03/23] |
|  | Michael agrees with this, saying that objective tests are more reliable and that they should limit subjective measures. Here he adds in that “of course, we need to stay in line with what everybody else is doing.” [observation, orthopaedic, 16/02/23] |
| ***Logistics of choice*** | Victor, who has been quiet for a while expresses concerns over the logistics of this stating “it’s not about the drink, it’s about the logistics of giving them the drink.” He raises the issue of timing of receiving the drink and the problems that this can cause for anaesthetists. [observation, orthopaedic, 16/02/23] |
| ***Climate of respect and collegiality influences discussions*** | There was ongoing collegial discussion and no conflict or heated debates. The group tended to listen and contribute but in a collaborative way that seemed to back up what others were saying. There was genuine enthusiasm to implement the pathways. [observation, spinal implementation, 10/10/23] |
|  | “In my limited experience in this role, I'll go to meetings […] And you know, there's there seems to be this reciprocated respect and a culture of voicing and sharing, collaborating.” [Int 8, clinician] |
| ***Determinants*** |  |
| ***Funding and resources*** | “Like we, we just haven't had the capacity kind of to investigate across disciplines the way that people are providing care and to kind of say, well, let's let's have this standard approach. Really, it's just we've accepted the approaches, that have been brought in, which are, often disparate.” [Int 1, management] |
| ***Institution priorities*** | Clinical standardisation is an ongoing goal for the Hospital as clinicians from disparate backgrounds bring their own practices or ‘flair’ to the hospital which the hospital tries to accommodate for however, too much can affect patient care and lead to unwarranted variation. [observation, cardiac, 6/03/24] |
|  | Harry continues on about how “our performance may not have been the same” as peer organisations and so it was in the interest of the organisation to start this work. [observation, breast surgery, 21/03/24] |
|  | During the introductions, he reiterates that “our focus is to do things better and then reduced length of stay will come.” [observation, orthopaedic, 16/02/23] |
| ***Institution type*** | “So it's kind of, this might be a historical viewpoint, is that private hospitals have adhered to this principle of practitioner um, autonomy and individuality, and have have accepted that there will be, um, ahh, individualised practise amongst the practitioners that work within their four walls. As opposed to like a public system where you've kind of got like a department and the department kind of oversees operations and there's a a lead to the department and that kind of dictates practise. In some ways, every individual who comes to operate at a private hospital is is the leader of their own practise… But there's still this this um, [pauses] a philosophical question about what is the role of the institution? Particularly a private institution, in leading, driving, um, providing systems for looking to reduce clinical variation to standardise care. Is, I think it's an ongoing question is is does a private facility have a role in doing that, or are you really just providing the walls within which people can can can flex their independent clinical practise? And you know that might be an enduring question particularly in um, just say, um, I should say corporatised private healthcare.” [Int 1, management] |
|  | “Yes, ah, it's more of the balance between yes, they are also our clients, but you know we're also business that we, we you have to adhere to our protocols.” [Int 7, clinician] |
| ***Individual variables*** | But does their responsibility extend beyond their own particular patients to a sort of a more Hospital or population based approach? [mumbles] I don’t know. They'll, they'll, they'll differ. And of course, you know people who have got responsibilities to other organisations and responsibilities to clinical practise and that whole kind of, you know, question of what? What? Time priority is. Is this of mine? What the Hospital is doing as an institution? Some people will prioritise that and and be able to dedicate time to that and other people will be dedicating that time and priority to other organisations where they work and and I dare say that just as the same in physiotherapy, you've got probably also people who are just predominately interested in in their own practise and the outcomes of their own patients. [Int 1, management] |
| ***Ready for behaviour change*** | Sarah says “that’s going to be a lot of buy-in from” from the cardiologists to transition to patients recovering in angiography and then being sent home. Enzo says “[Surgeon X] has bought-in” to which Sarah agrees but then says “others may be more old school in their thinking and would want the patients ‘babysat’ for a little while.” [observation, cardiac, 24/04/24] |
|  | “You got like say 5 anaesthetists do 60% of the practice, you might have a tail end of 15 anaesthetists that just to do the remaining 40% of the cohort. Each of whom are more individuals rather than necessarily being part of a broader group. Well then it's it's, it's, tricky to influence those people. How do you influence them? Well, like perhaps on people like the discipline lead for anaesthetics to say, look, we really want to move towards this approach. Can you come inside the tent and be part of that, you know, group that agrees to practise in this way?” [Int 1, management] |
|  | Harry mentions that one surgeon involved in the pathways development said to encourage behaviour change with surgeons, you need to show them where they sit individually on the scale as other surgeons are only interested in data if you show people their individual results rather than the discipline overall. Everyone nods in agreement. [observation, spinal implementation, 10/10/23] |
| ***Value of pathways*** | Harry then shows Curtis the potential outputs that the organisation can produce to show how the surgeons are tracking. Curtis is excited about this as Harry explains that the plan is to produce 3 monthly datasets on outcomes such as length of stay, intensive care unit admissions, patient demographics etc related to episodes of care. [observation, spinal, 17/08/23] |
|  | “I think people are coming to the meeting understanding that, you know, we are trying to streamline things and I think that's better.” [Int 4, clinician] |
|  | Nancy brings the conversation back to “what is the purpose of the pathway?” and says the point is “to make everyone’s life easier isn’t it?” and I find the different perceptions of uses for clinical pathways and the benefits interesting. Harry says yes (but appears to agree only to an extent) and explains how the purpose is to reduce clinical variation overall and in doing so, may result in other benefits such as decreased documentation. [observation, spinal implementation, 10/10/23] |
| ***Pre-condition: Goals align creating a climate for consensus*** | Nancy comments “we’re all working on the same thing” [observation, spinal implementation, 15/11/23] |

Abbreviations: CEC, Clinical Excellence Commission; DVT; Deep vein thrombosis; ICU; Intensive care unit; Int, interview; PAC; preadmission clinic; PROMs, patient reported outcome measures; PREMs, patient reported experience measures.

Note: quotations have been de-identified, and any names used are pseudonyms and not any participant’s real name.
